# High Prevalence of *inh*A Promoter Mutations among Patients with Drug-Resistant Tuberculosis in KwaZulu-Natal, South Africa

**DOI:** 10.1371/journal.pone.0135003

**Published:** 2015-09-02

**Authors:** Abraham J. Niehaus, Koleka Mlisana, Neel R. Gandhi, Barun Mathema, James C. M. Brust

**Affiliations:** 1 Department of Medical Microbiology, National Health Laboratory Services and University of KwaZulu-Natal, Durban, South Africa; 2 Departments of Epidemiology, Global Health, and Medicine, Rollins School of Public Health, Emory University, Atlanta, Georgia, United States of America; 3 Department of Epidemiology, Columbia University Mailman School of Public Health, New York, New York, United States of America; 4 Department of Medicine, Albert Einstein College of Medicine and Montefiore Medical Center, Bronx, New York, United States of America; St. Petersburg Pasteur Institute, RUSSIAN FEDERATION

## Abstract

**Background:**

Drug-resistant tuberculosis (TB) remains extremely difficult to treat because there are often few remaining active medications and limited diagnostic options to detect resistance. Resistance to isoniazid is typically caused by mutations in either *kat*G or the *inh*A promoter. *inh*A mutations confer low-level resistance to isoniazid and cross-resistance to ethionamide while *kat*G mutations confer high-level isoniazid resistance and no cross-resistance. Line Probe Assays (LPAs) that detect mutations in *kat*G and *inh*A are currently performed on all positive TB cultures in KwaZulu-Natal province, South Africa, but the frequency of *inh*A mutations in drug-resistant TB patients has not been examined.

**Methods:**

We sought to determine the proportion of patients who could potentially benefit from high-dose isoniazid and who may be resistant to ethionamide. We reviewed 994 LPA (Hain MTBDR*plus*) results at the TB reference laboratory in KwaZulu-Natal to determine the frequency of mutations in either *kat*G or the *inh*A promoter. We stratified these results by drug-resistance category (i.e., MDR-TB, pre-XDR-TB, and XDR-TB) as determined by phenotypic drug-susceptibility testing.

**Results:**

Among MDR- and XDR-TB isolates, the prevalence of *inh*A mutations without a concurrent *kat*G mutation was 14.8% and 10.3% respectively. The prevalence of *inh*A mutations with OR without a *kat*G mutation was 30.3% and 82.8%, respectively.

**Conclusion:**

More than 10% of patients with MDR- and XDR-TB may benefit from high-dose isoniazid. Although ethionamide is empirically included in all MDR- and XDR-TB regimens, nearly a third of MDR-TB patients and a majority of XDR-TB patients likely have resistance to ethionamide. Laboratories performing line probe assays should report specific band patterns so that clinicians may adjust treatment regimens accordingly.

## Introduction

South Africa has one of the world’s worst epidemics of multidrug-resistant (MDR) and extensively drug-resistant (XDR) tuberculosis (TB). The incidence of MDR-TB in South Africa has increased 5-fold since 2002 and there were 26,000 cases reported in 2013 [1]. Management of MDR- and XDR-TB is challenging, in part, because although treatment guidelines advise using a minimum of 4 active medications [2], there are often few remaining active drugs to which the patient’s isolate is susceptible.

Although one gene is responsible for ~95% of rifampin (RIF) resistance, resistance to isoniazid (INH) is generally due to mutations in one of two major genes: *kat*G or the *inh*A promoter. Mutations in *kat*G typically result in high-level INH resistance but do not generate cross-resistance with any other known TB medication [3]. In contrast, *inh*A promoter mutations produce low-level resistance to INH and, additionally, confer high-level cross-resistance to ethionamide, a second-line TB medication used in most MDR- and XDR-TB regimens [4–7]. The issue of cross-resistance is critically important to patients with drug-resistant TB as treatment often relies on the empiric use of second and third-line anti-TB drugs with poor efficacy and tolerability. The availability of any additional active medications can significantly improve treatment outcomes.

Use of fewer than 4 drugs or use of drugs to which the isolate is resistant risks treatment failure and generation of further resistance. If patients with MDR-TB are resistant to ethionamide but treated with a regimen containing it, the combination may not be sufficient to prevent emergent resistance to other medications in the regimen—possibly resulting in XDR-TB. In South Africa, susceptibility testing to ethionamide is not routinely performed, but in patients with MDR-TB, ethionamide is usually assumed to be active and included in all regimens because most patients have never previously received the drug. Ethionamide, however, adds no benefit to a patient with an *inh*A promoter mutation, and may, in fact, cause considerable toxicity, such as nausea and hypothyroidism. High-dose INH has been shown, in some studies, to add considerable benefit to patients with MDR-TB and low-level INH resistance [8, 9]. Identification of those patients who might benefit from high-dose INH could dramatically improve treatment outcomes for some patients with MDR- or XDR-TB. Patients with a *kat*G mutation, however, would not be expected to benefit from high-dose INH.

The practical utility of knowing which gene is mutated for a given patient depends on how common *inh*A mutations are in a given setting. The province of KwaZulu-Natal was the site of the first reported XDR-TB outbreak [10] and remains the epicenter of the drug-resistant TB epidemic, with more than 6000 cases of MDR-TB reported in 2012 [11]. If a substantial proportion of patients have isolates with an *inh*A promoter mutation, then a large number could potentially benefit from high-dose INH if the providers know which patients have that mutation. Availability of such information will also reduce ineffective use of ethionamide in these patients.

Line probe assays (LPAs) are rapid, genotypic tests of *Mycobacterium tuberculosis* (MTB) resistance. LPAs use polymerase chain reaction to amplify specific resistance-determining regions/genes from MTB DNA present in the sputum sample or culture isolate. The assays detect mutations in the most common resistance genes for INH and RIF (i.e., *kat*G & *inh*A, and *rpo*B respectively) and provide results in approximately 5 hours. In KwaZulu-Natal, LPAs were added to the TB diagnostic algorithm in 2009 and all positive cultures are now routinely tested with an LPA to screen for genotypic resistance to either INH or RIF.

To determine the proportion of patients who might 1) benefit from high-dose INH and/or 2) harbor resistance to ethionamide, we retrospectively reviewed the results of LPAs performed at the TB reference laboratory for KwaZulu-Natal province and calculated the relative proportions of patients with *kat*G and/or *inh*A promoter mutations.

## Methods

### Setting

KwaZulu-Natal province has a population of 10.5 million and a TB incidence of 897 cases per 100,000 population [12]. There is a single TB reference laboratory for the province where all mycobacterial cultures and drug-susceptibility testing are performed. LPAs (MTBDR*plus*, Hain Lifesciences) are performed on all positive cultures to rapidly identify drug resistance, and are performed in accordance with the manufacturer’s guidelines. All isolates with genotypic resistance to either INH or RIF undergo phenotypic agar proportion drug-susceptibility testing (DST) to INH (1 mg/L), RIF (2 mg/L), streptomycin (2 mg/L), kanamycin (16 mg/L) and ofloxacin (2 mg/L). Until recently, LPA results were sent to the treating clinician and reported as either “resistant” or “susceptible” to either drug but the specific band pattern (i.e., mutation results) were recorded only at the laboratory. The lab performs approximately 13,000 liquid TB cultures and 2000 LPAs each month.

### Data Collection

We reviewed 1000 routine LPA results performed at the laboratory from July 2012 to July 2013; we selected a group of consecutive results from each month of the year to account for possible seasonal variation or a single cluster (mean 76±13 samples per month). Isolates were eligible for inclusion if they showed resistance in any of the three genes tested by LPA. We recorded the specific polymorphisms in each gene as well as the results of phenotypic DST. Using the phenotypic DST as the gold standard, we categorized each isolate as MDR-TB (resistance to only INH plus RIF), pre-XDR-TB (resistance to INH, RIF and either ofloxacin *or* kanamycin, but not both), or XDR-TB (resistance to INH, RIF, ofloxacin *and* kanamycin).

If multiple isolates were identified from the same patient, only the first isolate was included and any isolates for which phenotypic DST results were not available were excluded.

### Analysis

To determine how many subjects might have been eligible for high-dose INH, we calculated the proportion of isolates containing an *inh*A promoter mutation but which did not have a *kat*G mutation, and stratified these results by drug-resistance category. Similarly, to determine the number of subjects who would not have benefitted from ethionamide, we calculated the proportion of isolates with an *inh*A mutation with or without a *kat*G mutation. Lastly, we examined the specific polymorphisms present in each gene to determine if any single locus was more or less common than others or associated with a particular resistance category.

### Ethics Statement

The study was approved by the Ethics Committees of the University of KwaZulu-Natal and Albert Einstein College of Medicine. These committees waived the requirement for informed consent as all data were previously collected during the course of routine medical care and the study did not pose any additional risks to the patients.

## Results

Among the 1000 patient isolates with available LPA results examined, 6 were missing phenotypic DST results and were excluded. The remaining 994 isolates represented all 11 districts of the province, drawing from 56 different hospitals and clinics.

Of the 994 isolates with phenotypic DST results, 70 (7.0%) had RIF mono-resistance and were also excluded from further analysis. Of the remaining 924 isolates 129 (14.0%) had INH mono-resistance, 694 (75.1%) were MDR-TB, 72 (7.8%) were pre-XDR-TB, and 29 (3.1%) were XDR-TB (Table 1). Twenty-one (2.3%) and 91 (9.8%) of isolates were classified by LPA as RIF mono-resistant and INH mono-resistant, respectively, though phenotypic DST showed them to be MDR-, pre-XDR- or XDR-TB and thus resistant to *both* INH and RIF. In these isolates, the LPA did not identify the resistance polymorphisms for INH and RIF respectively. One isolate was incorrectly classified by LPA as RIF-resistant when the phenotypic DST showed susceptibility and one isolate was incorrectly classified by LPA as INH-resistant when DST showed susceptibility.

**Table 1:** Results of line probe assay testing by phenotypic drug susceptibility testing result.

		LPA Results (mutation present)							
		INH Mono-resistance			RIF Mono-resistance	INH and RIF resistance			
		*inh*A only	*kat*G only	*inh*A & *kat*G	*rpo*B only	*rpo*B & *inh*A	*rpo*B & *kat*G	*rpo*B & *inh*A & *kat*G	TOTAL
**Culture DST Results**	INH Mono-resistance	21	89	15	1	1	2	0	**129**
	RIF Mono-resistance	0	1	0	68	1	0	0	**70**
	MDR-TB	4	28	38	21	99	435	69	**694**
	Pre-XDR-TB	0	3	6	0	3	35	25	**72**
	XDR-TB	0	1	11	0	3	4	10	**29**
	**TOTAL**	**25**	**122**	**70**	**90**	**107**	**476**	**104**	**994**

INH: isoniazid; RIF: rifampin; LPA: line probe assay; MDR: multidrug-resistant; XDR: extensively drug-resistant tuberculosis. TB: tuberculosis; DST: drug-susceptibility test

**Fig 1:**
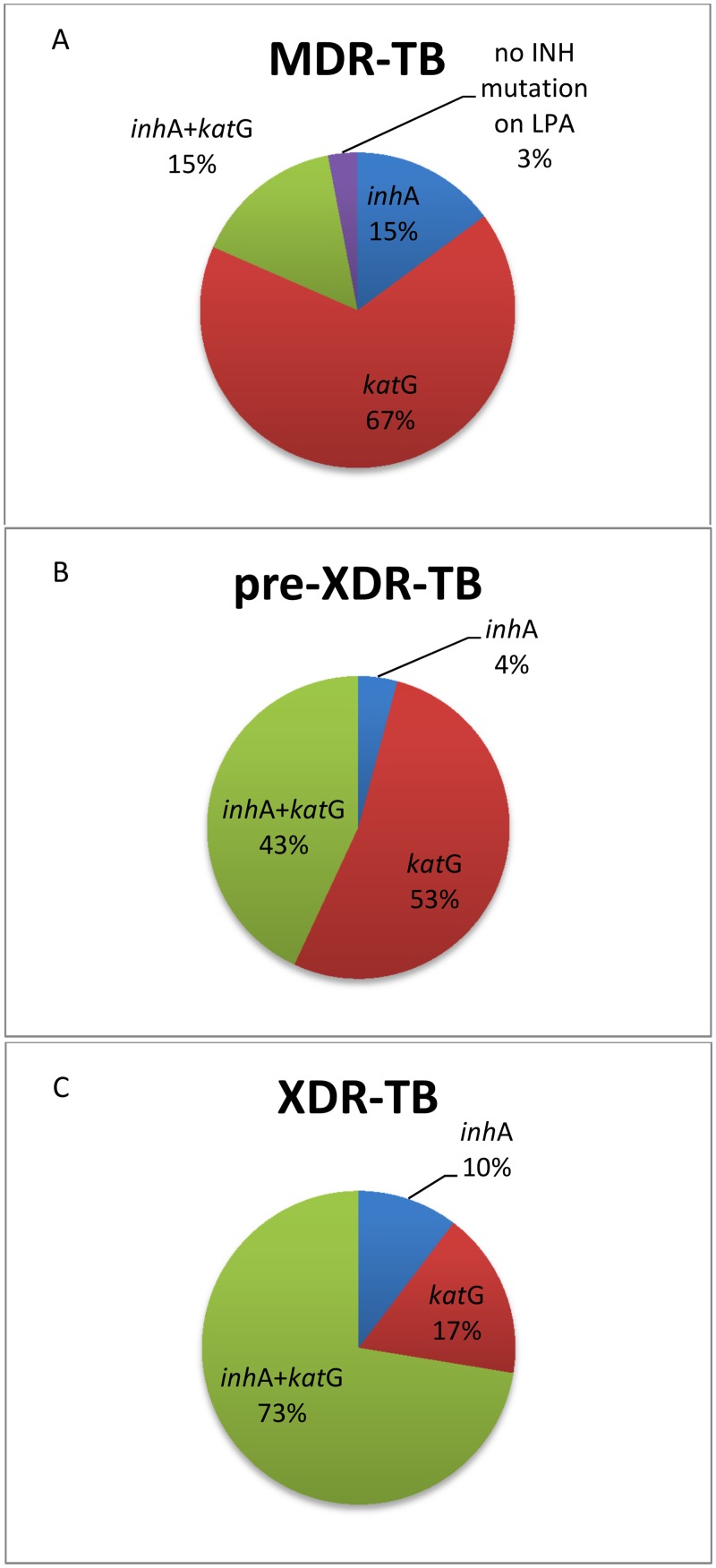
Proportion of isolates with *kat*G and/or *inh*A promoter mutations by drug resistance category.

Overall, 132 (14.2%) isolates had an *inh*A promoter mutation without a concurrent *kat*G mutation, representing 14.8% of those with MDR-TB, 4.2% of those with pre-XDR-TB and 10.3% of those with XDR-TB (Fig 1). These patients could potentially benefit from the addition of high-dose INH to their treatment regimen. Three hundred and six (33.1% of 924) isolates had an *inh*A mutation with OR without a *kat*G mutation, representing 30.3% of those with MDR-TB, 47.2% of those with pre-XDR-TB, and 82.8% of those with XDR-TB (p<0.001 for trend). These patients are likely resistant to ethionamide.

Specific polymorphism data was available for 754 (75.9%) isolates. Among the 232 isolates with an *inh*A mutation (regardless of *kat*G), 165 (71%) had the -15C→T mutation and the remaining 67 (29%) had the -8T→A mutation. No isolates carried either the -16A→G or the -8T→C polymorphisms (Table 2).

**Table 2:** Specific inhA promoter mutations found in each drug-resistance category.

Mutation	-15C/T	-16A/G	-8T/C	-8T/A	Total
**DST Category**					
INH Mono-Resistance	19 (54%)	0	0	16 (46%)	**35**
MDR-TB	123 (75%)	0	0	40 (25%)	**163**
Pre-XDR-TB	21 (78%)	0	0	6 (22%)	**27**
XDR-TB	2 (29%)	0	0	5 (71%)	**7**
**TOTAL**	**165 (71%)**	**0**	**0**	**67 (29%)**	**232**

INH: isoniazid; DST: drug susceptibility testing; MDR: multidrug-resistant; XDR: extensively drug-resistant

## Discussion

This is the first study to examine the prevalence of *inh*A promoter mutations among a large population of patients with drug-resistant TB in KwaZulu-Natal. We examined results of routinely-performed LPAs over a one-year period from the single reference lab in the province and found that more than 10% of patients with MDR-, pre-XDR-, and XDR-TB have *inh*A promoter mutations without a concurrent *kat*G mutation. These data underscore the need to clarify the effectiveness of high-dose INH, as a large number of patients in KwaZulu-Natal could potentially benefit if the efficacy of this therapy is confirmed. Indeed, some practitioners in South Africa have already started using high-dose INH out of desperation for patients with XDR-TB, but this is often in the absence of knowing the specific resistance mutations.

Several recent reports have examined relative frequencies of *kat*G and *inh*A promoter mutations in INH-resistant isolates and have shown considerable geographic variation, with 0.8% frequency of *inh*A (without *kat*G) mutation in Ethiopia [13], 4% in Poland [14], 9.6% in Chonqing, China [15], and 22% in the Philippines [16]. In Saudi Arabia, a study of isolates from 9 regional centers found that *inh*A mutations were more common in Southeast Asian immigrants compared to both the autochthonous population as well as African immigrants [17]. A study from the Western and Eastern Capes of South Africa found that nearly 40% of MDR-TB isolates carried an *inh*A promoter mutation without concurrent *kat*G mutation [18], but there is considerable genetic diversity of TB throughout South Africa and this precludes extrapolating results between provinces [19].

INH is a highly potent and rapid killer of MTB. The discovery that different genetic polymorphisms cause different levels of INH resistance could have important implications in the management of MDR- and XDR-TB. If low-level resistance to INH can be overcome with higher doses of INH, then this would add a much-needed effective drug to otherwise weak regimens for MDR- or XDR-TB. To date, the evidence for using high-dose INH is promising, but sparse. One randomized, three-arm, controlled trial in India compared high-dose INH, normal-dose INH or placebo plus an optimized background regimen for MDR-TB.[8] Patients who received high-dose INH achieved culture conversion significantly faster than those in the other two groups. There was no statistically significant difference in the overall proportion converting by 6 months, but this was limited by the small sample sizes in each group. One recent observational study found that a novel, short-course MDR-TB regimen containing high-dose INH (along with a regimen containing gatifloxacin, kanamycin, clofazimine, prothionamide, ethambutol and pyrazinamide) achieved excellent treatment outcomes with only 9 months of therapy, but this study lacked a control arm [9].

In our study, we also found that a large number of patients had an *inh*A promoter mutation with or without a concurrent *kat*G mutation, signifying that these patients are likely resistant to ethionamide. Because ethionamide is included in most MDR- and XDR-TB regimens, patients with ethionamide resistance are unnecessarily subjected to its considerable toxicity.

The frequency of *inh*A promoter mutations increased as the overall drug-resistance of the isolate increased, from MDR-TB to pre-XDR-TB to XDR-TB, but this could be due to increased clonality from transmitted strains. We have previously shown that TB strain diversity (as measured by spoligotyping) decreases with increasing drug-resistance in KwaZulu-Natal [20].

This study has several limitations. This was a retrospective study of laboratory records of routine testing. The original TB isolates were not available and we were thus unable to measure minimum inhibitory concentrations for INH to precisely correlate the LPA genotypic results with high or low-level phenotypic resistance. Similarly, we were unable to perform phenotypic resistance testing to ethionamide to confirm cross-resistance in those with an *inh*A promoter mutation. Since the original discovery of *inh*A, however, many groups have found an association between low-level INH resistance and cross-resistance to ethionamide [5]. Secondly, we did not sequence any of the other minor genes associated with INH-resistance (e.g., *ahp*C, *kas*A, *ndh*, *nat* and *msh*A). While almost all isolates in our study had a *kat*G or *inh*A promoter mutation, it is possible that some of those with only an *inh*A promoter mutation had an additional, undetected mutation elsewhere, conferring high-level INH resistance and obviating the potential benefit of high-dose INH. The Hain LPA, however, was specifically designed to capture only *kat*G and *inh*A because mutations in these two genes represent the overwhelming majority of INH resistance from diverse geographic areas.

KwaZulu-Natal is the largest province in South Africa and has the highest number of both TB and drug-resistant TB cases in the country. Mortality for drug-resistant TB remains exceptionally high and while new drugs and diagnostic tests may help improve outcomes in the long term, the National TB program, and programs around the world will need to optimize the use of existing medications in the short term. Our data have immediate implications for changing practice in KwaZulu-Natal, but also suggest that other countries with drug-resistant TB should conduct similar surveys and adjust diagnostic and management strategies accordingly.

LPAs provide rapid and actionable information for health providers of patients with drug-resistant TB. Given the high prevalence of *inh*A promoter mutations among patients with drug-resistant TB in certain settings, providing specific gene mutations when reporting LPA results may allow clinicians to adjust a patient’s regimen and maximize its effectiveness. To that end, the reference laboratory in KwaZulu-Natal province recently began providing specific gene mutations with LPA results. Further studies are required to determine the true effectiveness of high-dose INH for patients with low-level INH resistance.
